# Asymmetries between achromatic and chromatic extraction of 3D motion signals

**DOI:** 10.1073/pnas.1817202116

**Published:** 2019-06-17

**Authors:** Milena Kaestner, Ryan T. Maloney, Kirstie H. Wailes-Newson, Marina Bloj, Julie M. Harris, Antony B. Morland, Alex R. Wade

**Affiliations:** ^a^Department of Psychology, University of York, YO10 5DD York, United Kingdom;; ^b^York Neuroimaging Centre, University of York, YO10 5DD York, United Kingdom;; ^c^School of Optometry and Vision Sciences, University of Bradford, BD7 1DP Bradford, United Kingdom;; ^d^School of Psychology and Neuroscience, University of St. Andrews, KY16 9JP St. Andrews, United Kingdom;; ^e^York Biomedical Research Institute, University of York, YO10 5DD York, United Kingdom

**Keywords:** 3D motion, binocular vision, color

## Abstract

Motion in depth (MID) can be cued by high-resolution changes in binocular disparity over time (CD), and low-resolution interocular velocity differences (IOVD). Computational differences between these two mechanisms suggest that they may be implemented in visual pathways with different spatial and temporal resolutions. Here, we used fMRI to examine how achromatic and S-cone signals contribute to human MID perception. Both CD and IOVD stimuli evoked responses in a widespread network that included early visual areas, parts of the dorsal and ventral streams, and motion-selective area hMT+. Crucially, however, we measured an interaction between MID type and chromaticity. fMRI CD responses were largely driven by achromatic stimuli, but IOVD responses were better driven by isoluminant S-cone inputs. In our psychophysical experiments, when S-cone and achromatic stimuli were matched for perceived contrast, participants were equally sensitive to the MID in achromatic and S-cone IOVD stimuli. In comparison, they were relatively insensitive to S-cone CD. These findings provide evidence that MID mechanisms asymmetrically draw on information in precortical pathways. An early opponent motion signal optimally conveyed by the S-cone pathway may provide a substantial contribution to the IOVD mechanism.

## Significance

Navigating a 3D world requires sensitivity to motion in depth. The human visual system extracts two stereoscopic cues to motion in depth but little is known about the visual pathways that support their computation. Here, we show that achromatic inputs drive a 3D motion signal computed from slow changes in binocular disparity. However, the other 3D motion signal, based on interocular velocity differences, may draw preferentially on information carried by the evolutionarily ancient koniocellular pathway. Paradoxically, this pathway is thought to be far less sensitive to both motion and stereoscopic depth, yet, we find evidence for its involvement in the extraction of motion through depth. Our findings demonstrate an unanticipated role for the koniocellular pathway in a fundamental perceptual mechanism.

Two binocular cues support our perception of motion in depth (MID) (1, 2). The first, changing disparity (CD), monitors increases and decreases in binocular disparity over time. An object in space stimulates anatomically distinct parts of the left and right retinae, and the horizontal offsets between these two retinal images—the binocular disparity—provide a strong depth cue. Temporal changes in this depth cue therefore signify MID.

The second cue, the interocular velocity difference (IOVD), is based on a comparison of binocular opponent motion vectors. As an object moves toward or away from the eyes, it generates motion vectors pointing in opposing directions between the eyes. Comparing the sign and magnitude of these motion vectors provides an estimate of the speed and angle of MID.

Although both cues coexist in the natural world, each are sufficient to generate an MID percept in isolation (3–11). Due to constraints placed on the disparity and velocity computations they depend on, CD and IOVD operate optimally across reasonably distinct spatial and temporal ranges (12) and thus may be subserved by dissociable neural mechanisms.

Recent neuroimaging studies have emphasized the role of the human medial temporal (hMT+) area in processing both CD and IOVD (9), while corresponding neurophysiological evidence has identified cells tuned to 3D motion direction in this area (13, 14). Although hMT+ integrates both motion and disparity cues (15–18), no evidence for cross-cue adaptation between CD and IOVD has been found (6). This implies that separate subpopulations of neurons are tuned to either CD or IOVD within a common network of areas (19).

Other work suggests differences between CD and IOVD processing, both in an extended network of regions outside the hMT+ as well as in the pathways that relay cues to the hMT+. By comparing the fMRI response to a CD-type stimulus against the response to a static disparity plane, an area anterior to hMT+, the putative cyclopean stereo motion (CSM) area, has been identified as the potential locus of stereo-defined MID processing (20). V3A and regions in the parietal cortex, including the intraparietal sulcus (IPS), have been identified in an electroencephalography study where responses to MID stimuli were mainly driven by disparity cues (21). Finally, it has been suggested that, while a direct motion pathway from V1 to hMT+ may subserve IOVD computations, an indirect, parallel pathway via V2 and V3 relays disparity cues from V1 to hMT+ (17). Thus, the network of areas involved in CD motion processing may extend beyond areas involved in IOVD processing. Disparity and velocity signals may reach common MID areas via different, parallel pathways.

Cortical mechanisms underlying CD and IOVD can be dissected in greater detail by drawing on the chromatic specializations and response dynamics of precortical pathways. Generally, motion processing is dominated by achromatic signals carried by the magnocellular (MC) pathway, which constitutes the majority of inputs to the MT+ (22). Some achromatic inputs may be conveyed by the parvocellular (PC) pathway (23, 24), whose inputs reach MT+ via V1 and V2 (22). MT+ also receives direct, subcortical inputs from the S-cone driven koniocellular (KC) layers of the lateral geniculate nucleus (LGN) (25), and there is substantial evidence that S-cone–isolating stimuli can convey an equivalent motion percept when differences in contrast sensitivity are accounted for (26–30).

The spatial resolution of S-cone signals is constrained from the front-end of the system, given the sparse tiling of the S-cones in the retina, and the lack of S-cones in the fovea (31). In the LGN, cells in the KC layers have comparatively large receptive fields (32–35), although fMRI and electrophysiological measurements indicate that the relationship between spatial frequency tuning and receptive field size may break down in V1 (36, 37). Because of these properties, we hypothesized that an early, low-resolution S-cone signal may be particularly suited to conveying the coarse retinal motion vectors that are necessary for computing IOVD. Indeed, it has recently been shown that an S-cone–isolating stimulus is able to induce a 3D motion after-effect generated by adapting to monocular 2D motion (38).

**Fig. 1.**
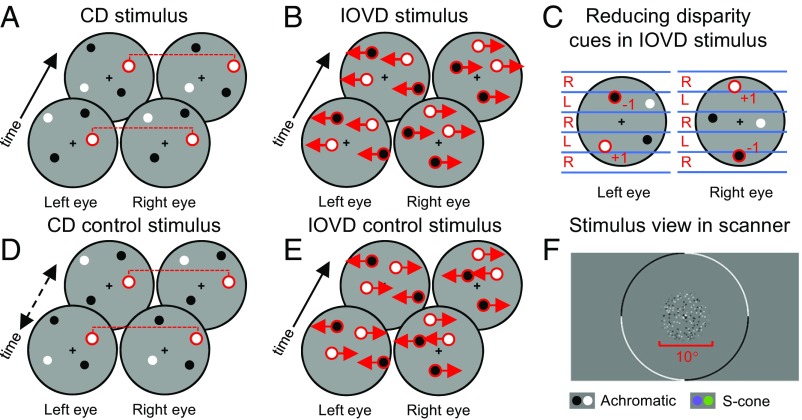
Stimulus design for the MID fMRI experiment. MID stimuli isolated mechanisms based on CD (*A*) or IOVD (*B*). Disparity cues were eliminated from the IOVD stimulus using a combination of decorrelation, anticorrelation, and spatial alternation of dot patterns in the left and right eyes (*C*). Control stimuli were matched to the MID stimuli on low-level properties but nulled the MID cue (*D* and *E*). All stimuli were presented with either achromatic or S-cone isolating dot patterns (*F*).

**Fig. 2.**
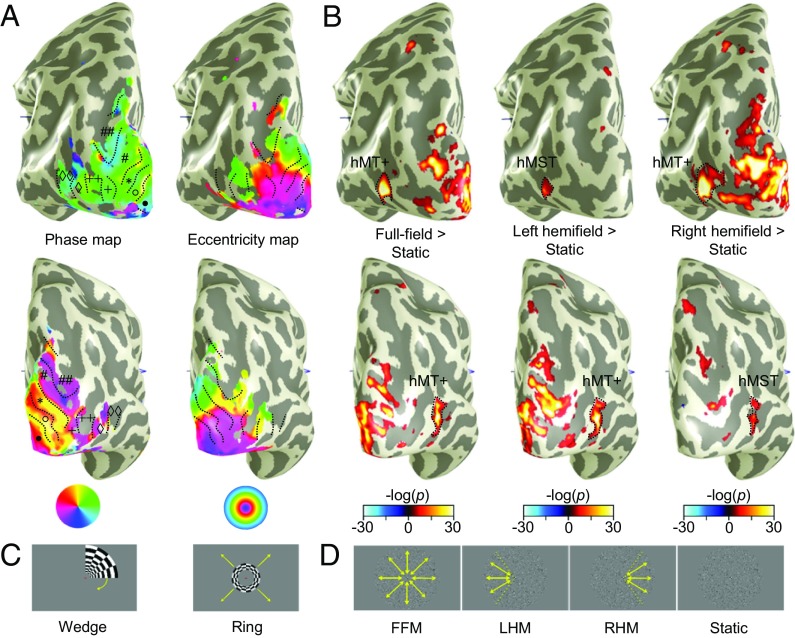
(*A* and *B*) ROI definition in an example participant in the left (*Upper*) and right (*Lower*) hemispheres. Symbols relate to ROIs drawn on the phase maps. V1 (•), V2 (°), V3 (*), V4, V3A/B (#), IPS-0 (##), LO-1 (+), and LO-2 (++) were defined based on the characteristic phase reversals in phase and eccentricity retinotopic maps (*A*, coherence threshold at 0.4), using periodically rotating wedge or expanding ring stimuli (*C*). hMT (⋄) and hMST (⋄⋄) were identified using motion localizers (*B*, threshold set at *P* > 0.00001) with full-field motion (FFM), left hemifield motion (LHM), right hemifield motion (RHM), and static stimuli (*D*). Contrasting LHM and RHM with static dots revealed subsets of voxels in hMT+ that were assigned to hMST in the ipsilateral hemisphere. Note that in the contralateral hemisphere, these contrasts often revealed a larger extent of activation than the FFM > static comparison in hMT+ (clearly seen in the *Upper* row of *B*).

Here, we used fMRI to probe the neural correlates of binocular MID perception. Stimuli were carefully designed to isolate the CD and IOVD cues, and for each stimulus type we generated matched control stimuli that nulled the MID cue (Fig. 1). Stimulus chromaticity was manipulated to investigate whether achromatic and S-cone pathways contribute to cortical CD and IOVD mechanisms differentially. Cross-fusible examples of the MID stimuli are provided in Movies S1–S4. fMRI results were analyzed at the group level, as well as individually in 11 predefined regions of interest (ROIs) spanning early visual areas, dorsal and ventral visual areas, and motion-sensitive areas of the cortex (Fig. 2). We measured psychophysical coherence thresholds in a subset of participants to relate fMRI findings to the perceptual detection of MID.

Based on previous neuroimaging research, we expected both cue types to engage motion pathways including areas hMT and human medial superior temporal (hMST) (6, 9), with possible additional CD responses in parietal areas and a stereo-motion area anterior to the hMST (20). Furthermore, we hypothesized that the S-cone pathway might be particularly suited to carrying the low-resolution motion signals required to compute IOVD, resulting in an S-cone advantage for perceiving IOVD-defined 3D motion.

## Results

### Psychophysics.

Participants’ sensitivity for detecting MID in achromatic or S-cone isolating CD and IOVD stimuli was assessed. To account for differences in cone contrast sensitivity between different precortical pathways and across early visual areas (39, 40), stimuli were contrast-scaled such that the contrast of the achromatic stimulus was one-tenth of the contrast of the S-cone stimuli. This ensured that stimuli were equally salient perceptually and should result in similar response amplitudes in neurons involved in detecting their presence (39) (*SI Appendix*, Figs. S1 and S2). Participants were required to indicate which stimulus interval contained MID in a two-alternative forced choice paradigm. The signal-to-noise ratio (SNR) of the MID stimulus was varied using a staircasing procedure, and threshold estimates were taken as the point at which participants could discriminate the MID stimulus from the control stimulus with 80% accuracy. Variance-weighted thresholds were computed for each participant, using four separate staircase procedures for each experimental condition (CD achromatic, CD S-cone, IOVD achromatic, and IOVD S-cone).

**Fig. 3.**
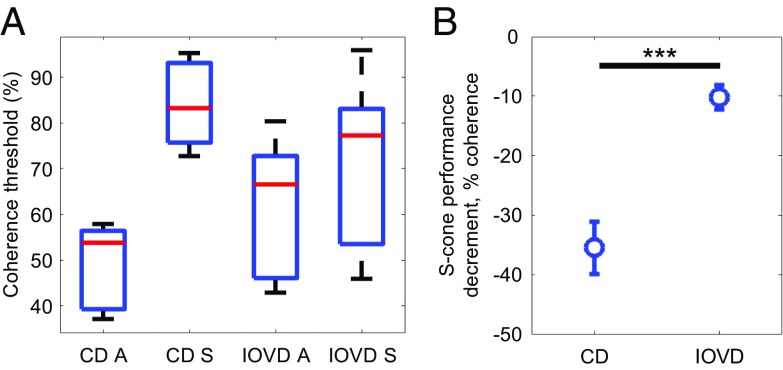
Psychophysical coherence thresholds measured for achromatic and S-cone isolating CD and IOVD stimuli. (*A*) The proportion of signal dots (percent coherence) required to detect MID at an 80% correct threshold. A variance-weighted mean threshold was calculated across four trials for each condition and each participant. Box-and-whisker plots show the spread of results across the group. (*B*) The S-cone performance decrement, given by the difference in percent coherence between achromatic and S-cone isolating conditions for CD and IOVD stimuli. For each participant, the S-cone threshold was subtracted from the achromatic threshold, and the mean difference was calculated across participants. Error bars are ±1 SEM. ***P < .001. Values less than 0 indicate that a higher SNR was required to detect MID when the stimulus was S-cone isolating, relative to when the stimulus was achromatic.

Threshold estimates across the group are shown in Fig. 3*A*. The data were entered into a 2 × 2 repeated-measures ANOVA, where within-subject variables were MID type (CD or IOVD) and chromaticity of the stimulus (achromatic or S-cone isolating). The ANOVA revealed no main effect of MID type [*F*(1, 6) < 0.005, *P* = 0.985, ε^2^ partial < 0.005]. In contrast, there was a significant main effect of chromaticity [*F*(1, 6) = 63.47, *P* < 0.001, ε^2^ partial = 0.91], where the mean threshold for achromatic stimuli was lower than the mean threshold for S-cone stimuli. This implies that, on the whole, participants were more sensitive to coherence differences between the MID and control stimuli when the chromaticity of the stimulus was achromatic.

Crucially, the ANOVA found a significant interaction between cue type and chromaticity [*F*(1, 6) = 45.36, *P* = 0.001, ε^2^ partial = 0.88]. As shown in Fig. 3*A*, the difference in coherence threshold between achromatic and S-cone stimuli is larger for CD stimuli than for IOVD stimuli. For CD, there is a large reduction in sensitivity (higher coherence threshold) when the stimulus is S-cone. This is not the case for IOVD.

To clarify this finding, an “S-cone performance decrement” was calculated for each individual participant. The variance-weighted mean thresholds for S-cone stimuli were subtracted from the thresholds for achromatic stimuli. The mean magnitude of this S-cone performance decrement across participants is shown in Fig. 3*B*. A paired-samples *t* test confirmed that the performance decrement for the CD cue is significantly greater than for the IOVD cue [*t*(6) = −6.74, *P* < 0.001], where participants required, on average, 35% more signal to detect CD MID when the stimulus was S-cone isolating rather than achromatic. For the IOVD cue, participants only required 10% more signal.

Overall, these results imply that, when differences in contrast sensitivity are accounted for, MID mechanisms draw on achromatic and chromatic signals differentially. Participants were far less sensitive to detecting CD MID when the stimulus was S-cone isolating, relative to when the stimulus was achromatic. For IOVD, participants were almost equally sensitive irrespective of the chromaticity of the stimulus.

### Whole-Brain fMRI Results.

**Fig. 4.**
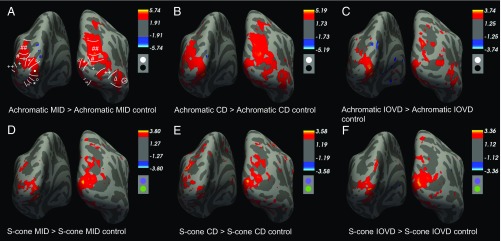
Results from the whole-brain analysis showing group-level *z*-statistic maps for responses to achromatic (*A*–*C*) and S-cone (*D*–*F*) MID stimuli. Dots below the scale bar illustrate the stimulus chromaticity. *A* and *D* show the combined responses to both CD and IOVD stimuli, compared with the combined response to CD and IOVD control stimuli. *B* and *E* show the CD response, and *C* and *F* show the IOVD response. (*A*) ROIs are: V1 (•), V2 (°), V3 (*), V4 (“), V3A/B (#), IPS-0 (##), LO-1 (+), LO-2 (++), hMT+ (Δ), and CSM (>).

Group results from the whole-brain, mixed-effects analysis are shown in Fig. 4. *Z*-statistic maps were generated by comparing the response to contrast-scaled MID stimuli against control stimuli. Control stimuli were designed to deliver similar local motion and disparity signals but no coherent MID. Stimuli were achromatic (Fig. 4 *A*–*C*) or S-cone isolating (Fig. 4 *D–F*). For Fig. 4 *A* and *D*, responses from both motion types (CD and IOVD) were taken together, while Fig. 4 *B*, *C*, *E*, and *F* show responses broken down by MID type and chromaticity.

A widespread network of areas involved in the computation of achromatic MID cues can be seen in Fig. 4*A*. This network includes visual areas V1, V2, V3, V3A/B, as well as ventral area V4, and motion-selective hMT and hMST. Activity extends dorsally to visually driven areas in the IPS, including IPS-0. In comparison, the network of areas involved in the S-cone MID response (Fig. 4*D*) is restricted to earlier visual areas, and responses in dorsal areas—such as IPS-0, hMT, and hMST—are weaker or absent.

The group maps hint at an interaction between the chromaticity of the input and the MID cue type. The achromatic MID response shown in Fig. 4*A* appears largely driven by the achromatic CD response shown in Fig. 4*B*, where the activation patterns are very similar. The achromatic IOVD map (Fig. 4*C*) is sparse; conversely, the S-cone IOVD response (Fig. 4*F*) is stronger and appears similar to the overall S-cone MID response (Fig. 4*D*). In this case, the S-cone CD response (Fig. 4*E*) is weaker than the achromatic CD response (Fig. 4*B*). To quantify these differences, an individual-level ROI analysis was carried out.

### ROI Results.

**Fig. 5.**
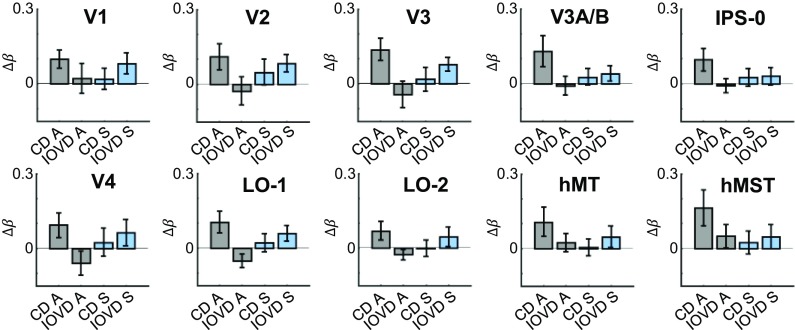
Results from the ROI analysis showing relative responses to different MID stimuli in a network of areas. Values on the *y* axis, Δβ, represent the differential response between MID stimuli and their respective control stimuli. Responses to achromatic CD (CD A), achromatic IOVD (IOVD A), S-cone CD (CD S), and S-cone IOVD (IOVD S) are plotted. The *y* axis values greater than zero represent a stronger response to the MID stimulus, and values less than zero represent a stronger response to the MID control stimulus. Error bars are ±1 SEM.

β-Weights representing the response to each of the nine stimulus conditions were extracted for each participant. Raw β-amplitudes to all stimulus conditions are plotted in *SI Appendix*, Fig. S2. The signal relating specifically to the MID cue was isolated by subtracting the modeled response to each control stimulus from the modeled response to each MID stimulus (Δβ), plotted in Fig. 5. These differences were entered into a 10 × 2 × 2 repeated-measures ANOVA modeling the response in 10 ROIs (V1, V2, V3, V3A/B, IPS-0, V4, LO-1, LO-2, hMT, and hMST) for two chromaticities (achromatic and S-cone) and two MID types (CD and IOVD).

We asked whether MID mechanisms can be driven equally by CD and IOVD cues. If both cues are extracted within a similar network of areas, as suggested by the group maps, we would expect both CD and IOVD stimuli to elicit a similar blood oxygenation level-dependent (BOLD) response in each ROI. In line with this, the ANOVA found no main effect of MID type [*F*(1, 10) = 1.46, *P* = 0.255, ε^2^ partial = 0.13, Greenhouse–Geisser correction for sphericity], implying that on average there was no difference between the effects of the two cues. In addition, we found no significant interaction between MID type and ROI [*F*(3.00, 26.66) = 0.67, *P* = 0.576, ε^2^ partial = 0.06, Greenhouse–Geisser correction for sphericity]. This indicates that, in line with our hypothesis, a similar network of areas is involved in computing MID for CD and IOVD mechanisms.

If these MID mechanisms were to be driven largely by achromatic inputs, we would expect to see a significant main effect of chromaticity or an interaction between chromaticity and ROI, because the response to achromatic MID stimuli would be higher in some or all ROIs. However, the ANOVA found no main effect of chromaticity [*F*(1, 10) = 0.06, *P* = 0.815, ε^2^ partial = 0.01, sphericity assumed] and no significant interaction between chromaticity and ROI [*F*(2.66, 26.67) = 2.08, *P* = 0.132, ε^2^ partial = 0.17, Greenhouse–Geisser correction for sphericity]. These results have two main implications: (*i*) that by contrast-scaling our stimuli, we succeeded in balancing the extent to which achromatic and S-cone inputs drive the BOLD response across ROIs, thereby avoiding bias by favoring either pathway; and (*ii*) that when this bias is avoided, MID mechanisms can be driven by both achromatic and S-cone inputs.

Thus, there appears to be no overall difference in the networks of areas involved in processing CD and IOVD, and no overall difference in the extent to which achromatic and S-cone information can contribute to MID. However, clearly, the sources of information—disparity and velocity—for both MID types are vastly different. Is there, then, a difference in the manner in which the early visual pathways convey these sources of information?

Crucially, the ANOVA revealed a significant interaction between MID type and chromaticity [*F*(1, 10) = 10.31, *P* = 0.009, ε^2^ partial = 0.51, sphericity assumed]. This indicates a dissociation of the chromatic inputs into the MID mechanisms. The CD response was larger when it was driven by achromatic input, but the IOVD response was greater when it was driven by S-cone input. This pattern was consistent across ROIs, implying that while the general network of areas involved in processing CD and IOVD are similar, the two cues can be differentiated on the basis of early chromatic inputs.

**Fig. 6.**
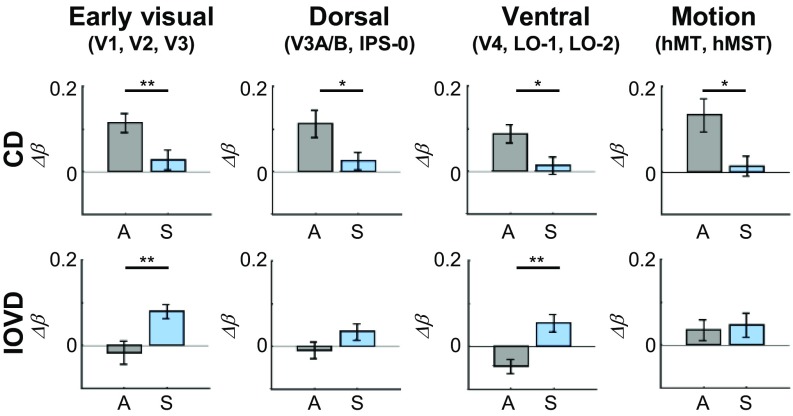
Results from grouped ROIs showing the difference between MID and control stimuli for CD (*Upper*) and IOVD (*Lower*) mechanisms. Responses to achromatic stimuli (A) are illustrated in gray, and responses to S-cone stimuli (S) are illustrated in blue. The *y* axis values (β-difference, Δβ= MID β-value − MID control β-value) greater than zero represent a stronger response to the MID stimulus, and values less than zero represent a stronger response to the MID control stimulus. Error bars are ±1 SEM. *P < 0.050, **P < 0.010.

To clarify this finding, results from different ROIs were averaged and grouped (Fig. 6). The β-differences (Δβ) calculated previously within each ROI were grouped into early visual areas (V1, V2, and V3), dorsal areas (V3A/B and IPS-0), ventral areas (V4, LO-1, and LO-2), and motion areas (hMT and hMST). Within each group of ROIs, differences between chromatic and achromatic stimuli for the same MID type were compared using a paired-samples *t* test.

Both CD and IOVD stimuli elicited reliable responses across all four grouped ROIs, but the amplitude of this response was dependent on chromaticity. For the CD mechanism, this response was driven by achromatic input (Fig. 6, *Upper*). For the IOVD mechanism, it was the S-cone stimulus that resulted in reliable responses (Fig. 6, *Lower*). Thus, although both types of MID are processed in a similar network, they appear to be optimally conveyed by different chromatic mechanisms.

The S-cone contribution to the CD response was weak in early visual and dorsal areas and negligible in ventral and motion-selective ROIs. Paired *t* tests comparing the S-cone and the achromatic CD responses showed stronger contributions from the achromatic pathway in early visual areas [*t*(32) = 3.10, *P* = 0.004], dorsal areas [*t*(21) = 2.17, *P* = 0.041], ventral areas [*t*(32) = 2.53, *P* = 0.017], and motion areas [*t*(21) = 2.78, *P* = 0.011].

In contrast to this, S-cone stimuli consistently elicited a stronger IOVD response than achromatic stimuli did, a pattern that was particularly striking in early visual and ventral areas. Here, paired *t* tests revealed significantly larger S-cone responses than achromatic responses [*t*(32) = −2.85, *P* = 0.008 for early visual areas, and *t*(32) = −3.44, *P* = 0.002 for ventral areas].

In comparison with this dominant S-cone input, achromatic contributions to the IOVD mechanism were weak. In early visual areas and in dorsal areas, the achromatic IOVD response was at zero. In ventral areas, the achromatic IOVD response was negative, implying that these areas respond more strongly to the control stimulus, which contained lateral motion energy but no MID. In fact, contributions from the achromatic pathway to the IOVD mechanism emerged only in the motion-selective ROIs, where stimulus-evoked responses were roughly equal regardless of chromaticity [*t*(21) = −0.36, *P* = 0.725].

### Analysis of the CSM Area.

**Fig. 7.**
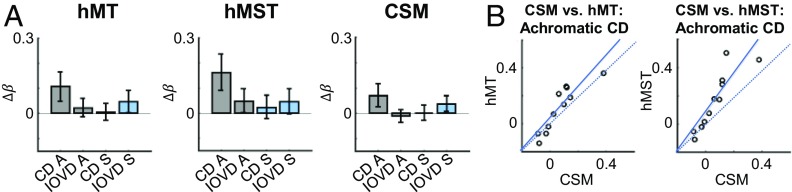
The CSM region of interest analysis. (*A*) Response patterns to achromatic CD, achromatic IOVD, S-cone CD, and S-cone IOVD stimuli in hMT, hMST and CSM ROIs. Values on the *y* axis are the difference in β-amplitude between MID and MID control stimuli (Δβ). (*B*) Correlations in response to achromatic CD between hMT and CSM, and hMST and CSM. The dashed line indicates a perfect correlation (*R* = 1), and data points are fitted with a least-squares line. Each data point represents responses for one subject. CSM responses were weaker than, but highly correlated with, responses in hMT and hMST.

Finally, we analyzed the activation pattern to achromatic and S-cone CD and IOVD stimuli in the putative CSM ROI. This region was first described by Likova and Tyler (20), who measured a strong CD motion response in an area anterior to the hMT and hMST. The CSM can be localized using Talairach coordinates provided in Likova and Tyler’s paper. Using this approach, we extracted β-weights for responses in the CSM ROI and found that overall response amplitudes were weaker than those measured in the hMT and hMST (Fig. 7*A*). Notably, the response to achromatic CD stimuli was significantly lower in the CSM than in hMST [*t*(10) = 2.77, *P* = 0.020, paired samples *t* test].

We found that in over half of all brains (6 of 11 participants used in the analysis), the CSM partially overlaps the hMST in at least one hemisphere. Overlaps with hMT occurred in three cases. The amplitude of the achromatic CD response (taken as the difference between the CD stimulus and the CD control stimulus) was highly correlated between the CSM and hMT (*R* = 0.89, *P* < 0.001) and the CSM and hMST (*R* = 0.88, *P* < 0.001). Correlation results are shown in Fig. 7*B*. Overall, we therefore found no evidence to suggest that the CSM is uniquely involved in CD processing, although populations of cells here may contribute to CD processing more generally in a manner analogous to cells in hMT+.

## Discussion

We used achromatic and S-cone–isolating random-dot stimuli that engaged CD or IOVD mechanisms to probe the neural pathways involved in MID processing. Broadly, we found that both CD and IOVD stimuli elicit BOLD responses in a network of areas that includes the early visual cortex, parts of the dorsal and ventral streams, and motion-selective areas. Because we measured no significant differences between these two cues overall, and no interaction between ROI and MID type, our findings are consistent with previous studies suggesting that signals for both cues are multiplexed in a common network of areas with different neural subpopulations tuned to either CD or IOVD (6, 41).

Our finding is that within this network, achromatic and S-cone signals contribute to a different degree to IOVD and CD. The CD cue appears to depend primarily on achromatic inputs, and S-cone CD responses were weak. Conversely, the S-cone IOVD stimulus elicited a strong response in several ROIs, including early visual areas and ventral areas. Achromatic IOVD responses were relatively weaker and began to emerge in later motion-selective ROIs.

We also measured psychophysical coherence thresholds to determine the effect of stimulus chromaticity on the detection of MID. Observers were far less sensitive to detecting CD MID when the stimulus was S-cone, in comparison with when the stimulus was achromatic. This was not the case for IOVD where, for our judiciously chosen sets of contrasts, participants were almost equally sensitive to stimuli of either chromaticity. Taken together, our findings suggest a critical dissociation in the way that early chromatic pathways contribute to CD and IOVD mechanisms.

### Achromatic and Chromatic Inputs to MID Mechanisms.

CD and IOVD responses measured across ROIs were dependent on the chromaticity of the stimulus. This interaction cannot be explained by overall differences in contrast; our stimuli were contrast-scaled such that on average there were no differences in the extent to which achromatic and S-cone signals drive activity across ROIs. Thus, achromatic signals conveyed by the MC and PC pathways, and S-cone signals conveyed by the KC pathway, both contribute to MID processing, although they contribute differentially to CD and IOVD.

Across all ROIs, the CD response was primarily driven by achromatic stimuli, while S-cone contributions were weak in early areas and negligible in motion-selective areas. Due to the low spatial resolution of the KC pathway, neural populations driven by S-cone inputs are limited in their ability to perform the highly precise spatial matching required to resolve fine retinal disparity. S-cones are able to provide inputs to disparity mechanisms through low spatial frequency channels only (42). Despite the weak cortical responses we measured here, our participants were able to perceive S-cone CD MID during the behavioral portion of the study, perhaps due to coarse disparity processing (43). Perceptually, participants were highly sensitive to achromatic CD, but relatively insensitive to S-cone CD, a finding that dovetails with the neural response profile. Our results suggest that the CD mechanism depends primarily on achromatic inputs with high spatial resolution.

In direct contrast to this, the IOVD signals we measured were biased toward S-cone inputs. We measured consistent BOLD modulations across ROIs to S-cone IOVD, while the achromatic stimulus appeared to contribute to IOVD mechanisms primarily in later, motion-sensitive areas. This may explain why previous fMRI research using achromatic IOVD stimuli has emphasized the role of hMT+ as the locus of IOVD processing (9).

Perceptually, participants were almost equally sensitive to achromatic and S-cone IOVD, with only a small performance decrement in the latter case. Together with our fMRI data, these findings suggest that the IOVD mechanism can be driven by both achromatic and S-cone inputs. Our findings of similar IOVD coherence thresholds for color and luminance inputs, as well as matched BOLD responses in motion-selective areas, are consistent with Shioiri et al.’s (38) observation that both color and luminance contribute to a velocity signal before the computation of IOVD. Our data indicate that the luminance drive to these IOVD inputs is, however, relatively weak compared with the luminance contribution to the CD system.

KC signals appear to be relayed particularly rapidly to extrastriate, motion-selective areas (44), suggesting an efficient mechanism through which the S-cones might contribute to motion processing. The precise source of S-cone signals in MT has been controversial. Direct anatomical projections from the KC layers of the LGN to MT have been used to explain sensitivity to moving isoluminant S-cone stimuli, measured perceptually (27) as well as with fMRI (45) and with electrophysiological recordings in the MT (46). Alternatively, S-cone signals may “piggyback” on the MC pathway, with some evidence suggesting S-cones input to around 10% of cells in the MC layers of the LGN (47). S-cone signals could in principle arrive at the MT through the dominant MC pathway input (48).

However, in this study we found an early dissociation in the extent to which achromatic and S-cone signals contribute to IOVD MID, suggesting at least two different underlying mechanisms. This dissociation was particularly evident in early visual and ventral areas. The differences were smaller in hMT+, implying a convergence of MC, PC, and KC signals in motion-selective ROIs.

We suggest that the large receptive field sizes in the KC layers of the LGN provide an early mechanism for computing IOVD-based MID. Because the IOVD cue depends on motion vectors generated at the level of the retina and does not necessarily require spatial matching between the eyes, it may integrate across larger portions of the visual field to generate reliable estimates of MID. Indeed, very sparse IOVD stimuli can convey MID percepts (49). Our findings suggest that early, low-resolution S-cone signals are combined in an opponent manner, and these signals contribute toward IOVD mechanisms through a network of ROIs.

### MID Signals in Primary Visual Cortex.

Both CD and IOVD stimuli elicited responses as early as V1 that were comparable in amplitude to those measured in later, motion-selective ROIs. Previous neuroimaging studies have reported only small responses here, with the strongest MID-driven responses recorded in hMT+ (9). There may be several reasons for this. First, our CD stimulus contained little depth context compared with the stimulus used by Rokers et al. (9), who divided their stimulus into quadrants moving in opposing directions. Our stimulus contained only the fixation point and the fixation ring, engaging more neurons tuned to absolute rather than relative disparity. This profile is consistent with the tuning properties of binocular disparity neurons in V1 but not of those further upstream (50), and may explain why we measured larger CD responses in V1 than previous studies.

Second, the CD control stimulus, which consisted of temporally scrambled frames from the CD stimulus, could lead to a more stochastic response from disparity-tuned neurons. In theory, two consecutive frames in the control stimulus can excite, then inhibit, a single neuron. This may not provide sufficient integration time for neurons to fire an action potential, leading to weak local field potentials and a weak BOLD response.

Classic motion energy models (51) would not predict a strong V1 response to IOVD motion, given that V1 cells have small receptive fields that are primarily tuned to component motion (15, 52) and do not exhibit strong motion opponency (53). Based on these properties, neural populations in V1 could provide early velocity estimates that are combined at a later stage to generate estimates of MID.

However, recent models of binocular motion perception in the MT suggest that V1 inputs should exhibit motion opponent suppression, and that these signals arise before binocular integration in V1 (54). A general, interocular suppressive mechanism may precede the extraction of MID (55), while monocular motion opponency has also been proposed to drive pattern motion cells in the MT (56, 57). There has also been some electrophysiological evidence for motion opponent suppression in V1, although these signals were weak, and it is unclear whether their source was monocular or binocular (58).

The IOVD responses we measured in V1 also suggest an early motion opponent signal. These signals could arise from joint motion and eye selective fields in V1, or early motion opponent inputs into binocular V1 cells. Crucially, this signal was larger for the S-cone stimulus than the achromatic stimulus, suggesting that dichoptic S-cone signals are combined in an opponent manner before V1. Some directionally selective cells in the KC layers of the LGN (59, 60) receive binocular inputs (61), and it has recently been suggested that the direction selectivity measured in blue-on cells in the KC layers of the LGN is generated by latencies between the “on” and “off” subfields of small bistratified ganglion cells in the retina (60). Such mechanisms could provide a very early basis for extracting the binocular motion-opponent signals in V1 that support IOVD.

### Other Areas Involved in the Extraction of 3D Motion.

The role of hMT+ in CD and IOVD processing has been documented previously (9, 13, 14), with emphasis on 2D and 3D motion being processed by the same cortical pathways (41). In addition to MID responses in classic motion pathways, from V1 to hMT+, we measured strong CD-driven responses in area IPS-0. The human IPS is involved in a variety of cognitive functions, including the top-down control of visual attention and eye movements, which modulates activity in earlier visual areas (62–64). In addition, the IPS also contains distinct populations of neurons that are sensitive to motion (65) and 3D structure from motion (66, 67). This may explain why activation in IPS-0 was more pronounced for CD stimuli than for IOVD stimuli. Because IOVD stimuli lack the concrete depth information provided by the binocular disparity cues in the CD stimulus (8), they are much less likely to convey shape or form information and are thus less likely to engage form-from-motion mechanisms. IPS-0 activation observed here may constitute a part of the MID pathway that is involved in extracting 3D shape from disparity and the allocation of visual attention, rather than in extracting 3D motion per se.

Previously, an area anterior to hMT+, the CSM area, has been suggested as the main locus for stereo-defined MID processing (20). We found no compelling evidence that this area is uniquely involved in the extraction of CD MID signals. Although we measured activation to the CD stimulus anterior to hMT+, activity was not restricted here, and we also measured strong modulations in hMT+ itself. Our ROI analysis of the CSM area showed a similar, but weaker, response profile to hMT and hMST across all stimulus types.

Unlike Likova and Tyler (20), we found substantial overlaps between the CSM ROI and our own hMST definitions, contributing to similarities in responses between these two regions. However, even in hMT, which was clearly distinct from the CSM in almost all participants, response profiles were very similar and the achromatic CD response was highly correlated between the CSM and hMT. We suggest that CD is extracted primarily in the hMT and hMST.

## Conclusions

We measured responses to CD and IOVD stimuli in a network of areas that included early visual areas, parts of the dorsal and ventral streams, and motion-selective hMT and hMST. Both achromatic and S-cone stimuli provided inputs to these areas, suggesting that signals carried in the MC, PC, and KC pathways all contribute to MID processing. However, we found that CD responses were most strongly driven by achromatic inputs, while the S-cone stimuli elicited only weak responses. This preference for achromatic inputs was also observed behaviorally, where there was a large decrement in sensitivity to CD when stimuli were S-cone isolating. For the IOVD cue, participants were almost equally sensitive to MID irrespective of input chromaticity. fMRI data showed that IOVD mechanisms across a hierarchy of areas were driven most strongly by S-cone inputs. Achromatic inputs generated a comparable response only in later, motion-selective ROIs. S-cone IOVD signals were robust even in V1, suggesting that KC signals are combined in an opponent manner at a very early stage in visual processing. Overall, we have shown that cortical CD and IOVD mechanisms asymmetrically draw on achromatic and S-cone signals within a shared network of areas.

## Materials and Methods

### Participants.

Participants (*n* = 17, aged 21–45 y, 7 male) with normal or corrected-to-normal vision were recruited. For the whole-brain analysis, data from all participants were used. For the ROI analysis, data from 6 participants were discarded due to poor fits in the general linear model (GLM < 5% variance explained across ROIs), leaving a final *n* of 11 for that analysis. Of these, seven participants were recruited for behavioral testing. Three participants were authors on this paper (M.K., K.H.W.-N., and A.R.W.); the rest were naïve. All participants had normal stereo-acuity [below 120 arcsec, measured using the TNO test, 19th ed (Laméris Ootech)] and normal color vision (tested using Ishihara plates, 24-plate edition).

Before scanning and behavioral testing, participants practiced the S-cone isoluminance setting task and viewed high-visibility exemplars of the MID stimuli. These were 100% coherent CD or IOVD stimuli oscillating continually in depth, with identical parameters to those shown during the experiment (described below). “Coherence” here refers to the SNR in the stimulus display, where in a 100% coherent stimulus all dots contribute to the MID signal. All participants reported a percept of oscillatory MID for all stimulus types. Written informed consent was obtained in accordance with the Declaration of Helsinki, and the study was approved by the York NeuroImaging Centre Board of Ethics.

### Apparatus.

For pretesting and behavioral testing, stimuli were displayed on a VIEWpixx 3D LCD system with 1,920 × 1,080 pixel resolution, running at 120 Hz, with a maximum luminance of 250 cd/m^2^. Stereo presentation was achieved using wireless NVIDIA GeForce 3D vision LCD shutter goggles and an infrared emitter that synchronized the frame rate of the display with the goggles (VPixx Technologies).

During scanning, a PROpixx DLP LED projector (VPixx Technologies) at 1,920 × 1,080 pixel resolution and running at 120 Hz was used to back-project stimulus images on to a silver screen positioned behind the participant. Stereoscopic stimulus presentation was achieved using a circular polarizer (DepthQ Polarization Modulator, VPixx Technologies) placed in front of the long-throw lens and passive 3D glasses worn by the participant. Stimuli were viewed on a first-surface mirror mounted on the head coil (57-cm viewing distance, including the optical pathway of the mirror), yielding a viewing angle of 41° × 23.5°. Maximum luminance, as measured through the polarizer and glasses, was 390 cd/m^2^.

Both display systems were photometrically calibrated using a fiber-optic photospectrometer (Ocean Optics) measuring the γ and the spectral irradiance of each R, G, and B channel as seen by each eye. The fiber-optic cable tip was positioned behind the goggles through a polystyrene mannequin head to match the participants’ viewing distance and position. Left and right eye measurements were taken, and as there were no significant differences between the eyes, an average was taken for color calibration.

Stimulus presentation during scanning and behavioral testing was controlled from a Shuttle PC with Intel Core i7-4790K processor at 4.0 GHz and an NVIDIA GeForce GTX970 graphics card with 4 GB DDR5 memory. All stimuli were designed and run from Matlab 8.5.0 (2015a; The MathWorks) in conjunction with Psychtoolbox 3.0.12 routines (68, 69). During scanning, participant responses and scanner trigger pulses to synchronize stimulus onset were transmitted using a fiber-optic response pad (Current Designs). During behavioral testing, participant responses were recorded using a keyboard.

### Stimulus Design.

We designed stimuli to isolate CD and IOVD cues independently (Movies S1–S4). We also generated appropriate null “controls” for each stimulus type that matched the low-level properties of the MID stimuli but conveyed no MID cues.

All stimuli were variants of dynamic random dot stereograms (70, 71), where antialiased dots were 0.5° in diameter presented within a cosine envelope that gradually smoothed the edges over 0.15°. Dot sizes were doubled for coherence thresholding to improve visibility of the stimulus. Dots were pseudorandomly positioned on a mean luminance gray background (390 cd/m^2^). The dot centers were at least 0.5° apart in any direction, and dots were assigned with a 0.5 probability to be either positive or negative contrast polarity: for achromatic stimuli, this was along the L+M+S color axis, and for S-cone stimuli, this was along the S-(L+M) color axis. S-cone dots were displayed at the maximum possible contrast given the display gamut (around 45% on both our systems). To balance the extent to which this cone contrast drives the BOLD signal in the early visual cortex (45), and the perceptual salience of achromatic and S-cone stimuli (27, 72) (*SI Appendix*, Figs. S1 and S2), the achromatic dot contrast was set to 10% of this value (4.5% Michelson contrast). The dynamic random dot stereograms were viewed through a circular aperture with edges smoothed by a Gaussian kernel [0.5° full-width half maximum (FWHM)] with a 0.5° inner and a 5° outer radius. A fixation cross (0.2° wide/high) was placed at the center of the annulus. Central (0.4° radius, centered around fixation) and peripheral (11.75° from fixation) achromatic fixation rings helped stabilize the MID percept. Stimuli were presented for 3 s with a cosine ramp to avoid fMRI signal transients, and the stimulus was at peak contrast for 1.5 s. A representation of the stimulus view is illustrated in Fig. 1*F*.

### CD Stimulus.

The CD stimulus generated an MID percept by systematically increasing and decreasing the binocular disparity between pairs of dots in the left and right eyes (Fig. 1*A*). The stimulus oscillated sinusoidally in depth at a frequency of 1.4 Hz, with a maximum of ±24 arcmin disparity (±12 arcmin shift per eye), well within the ±32 arcmin fusional limits of the achromatic and S-cone disparity mechanisms (42). The location of each pair of dots was refreshed with each frame (refresh rate 120 Hz) with a monocular density of one dot per square degree, eliminating any coherent lateral motion (or IOVD) from the stimulus. To ensure robust behavioral thresholds in the psychophysical experiments, the position refresh rate was decreased by a factor of 4 to improve visibility. Dots regenerated at the same dot position on four successive frames, but the rate of disparity change over time was the same for scanning and behavioral testing. In all cases, the stimulus was perceived as a plane of dots oscillating sinusoidally through depth.

For the fMRI sessions, the CD stimulus was shown at 100% coherence where all dots contributed to the MID signal. During psychophysical testing, the coherence of the stimulus was adjusted using a Bayesian staircase procedure (73). Noise dots introduced into the CD stimulus were identical in physical parameters to the CD signal dots but were positioned randomly in the left and the right eyes to disrupt the binocular disparity cue. Random matches between left and right eye “noise” dots may result in spurious depth cues but could not contribute to the smooth changes in disparity over time that generate the MID signal.

### CD Control Stimulus.

Individual frames in the pregenerated 100% coherent CD stimuli were shuffled over time (Fig. 1*D*), as per Rokers et al. (9). This preserved the binocular disparity information in each frame, but eliminated the smooth changes in disparity over time that generate MID. Thus, on average, the CD control stimulus contained the same range of binocular disparities but did not convey MID.

### IOVD Stimulus.

The IOVD stimulus consisted of dots that were moving in opposite directions between the left and right eyes, creating motion signals in each eye that were equal in magnitude but opposite in direction (Fig. 1*B*). Dot patterns were unpaired (“decorrelated”) between the eyes, with a monocular dot density of one dot per square degree. The stimulus oscillated sinusoidally in depth at a frequency of 1.1 Hz with a maximum lateral shift of ±200 arcmin between the eyes [±100 arcmin monocular horizontal displacement, giving a monocular dot velocity range of 0–1.7°/s to match the peak of the IOVD velocity sensitivity curve measured psychophysically (12)]. Each dot had a maximum lifetime of 50 ms, and visual transients were balanced by regenerating the same number of dots in new locations in each video frame. The perceptual quality of the IOVD stimulus was of a cloud of dots oscillating toward and away from the observer, with no concrete sense of position in depth due to the lack of depth-from-disparity cues (8).

A significant challenge in designing IOVD stimuli is to eliminate the possibility of binocular matches that could result in CD “leakage” (74). Previously, this was achieved by anticorrelating the contrast polarity of binocular dot pairs (6, 8, 9, 12, 75), degrading the disparity cue (76–78). Alternatively, left and right eye displays can be divided into “stripes,” where dots are presented in alternating bands in the left and right eyes (10, 79). Finally, dot patterns can be decorrelated between the left and right eyes (7).

We combined all three of these approaches (Fig. 1*C*). Displays were divided into stripes, and decorrelated dot patterns were shown in alternate stripes between the two eyes. If two dots fell in close proximity at the borders of these stripes, their contrast polarity was anticorrelated. In this manner, the CD cue was effectively eliminated in the IOVD stimulus.

A 100% coherent IOVD stimulus was presented during scanning. During behavioral testing, the SNR was varied (see *Psychophysics*, below). Because the IOVD signal depends on dots moving in opposite directions between the eyes, noise was introduced to the stimulus by equal numbers of dots moving both leftward and rightward in each eye. Thus, noise dots generated the same motion energy as signal dots, but by balancing leftward and rightward motion between the eyes the MID signal was nulled. Noise dots were perceived as flat, lateral motion with no oscillation through depth.

### IOVD Control Stimulus.

The control for the IOVD stimulus contained the same lateral motion energy as its counterpart but did not convey any MID. Dots moved in both directions within a single eye, nulling the binocular opponent motion signal that generates MID (Fig. 1*E*). All other aspects of the stimulus were identical to the IOVD stimulus.

### Isoluminance Setting.

Stimuli were specified initially in LMS cone-excitation space. Matrices for the conversion from LMS to RGB values were computed from the Stockman and Sharpe (80) 10° fundamentals for the L-, M-, and S-sensitive cones, and the spectral power distribution of the RGB phosphors for each eye. Because there are significant individual differences in macular pigment density, S-cone stimuli were adjusted to each participants’ subjective point of isoluminance using heterochromatic flicker photometry (81). This was performed in situ*,* before the commencement of scanning as well as behavioral testing. Participants viewed a field of dots presented to either the left or the right eye. Dots alternated at 7.5 Hz between positive (violet) and negative (lime) contrast polarity along the S-(L+M) color axis. Within each run, participants made small adjustments to the amount of L+M contamination until the minimum amount of flicker was perceived. Dots had a circular profile (0.5° diameter) and were positioned pseudorandomly with a density of one dot per square degree, where each dot center was separated by at least 0.5°. The field of dots was viewed through a hard-edged annular window with a 1° inner radius around fixation and a 6° outer radius. Dot position was refreshed with each left or right eye trial but stayed the same for each set of adjustments made by the participant. Participants completed three sets of adjustments for each eye separately. The average isoluminance setting for each participant and in each eye was used to specify the S-cone dots in MID and control stimuli.

### Psychophysics.

Participants’ sensitivity to MID in achromatic and isoluminant S-cone CD and IOVD stimuli was measured using dot coherence thresholds. The SNR in MID stimuli was incremented in a Bayesian ψ staircasing procedure (73), within a two-interval forced choice paradigm similar to other MID studies (4, 82). Participants indicated which of the two intervals contained MID, where one interval contained the CD or IOVD stimulus and the other contained the respective control stimulus. The staircase estimated the α- (threshold) and β- (slope) parameters of a fitted Weibull function, where the threshold was taken as the percent coherence required for participants to correctly discriminate the MID stimulus with 80% accuracy. Dot coherence (SNR) varied between 0 and 100% in steps of 1%, referring to the proportion of dots that contributed to the MID percept.

Each trial was preceded by the fixation lock and fixation mark, before presentation of the two stimulus intervals. Participants pressed “1” or “2” on the keyboard to indicate the MID interval. Feedback was provided by presenting “correct” or “incorrect” for 500 ms after the response. CD and IOVD, as well as chromaticity, were tested in separate runs. Runs consisted of two interleaved staircases with 30 stimulus pairs in each staircase. One practice run followed by two test runs were completed for each participant and each stimulus condition, yielding a total of four threshold and slope estimates per condition.

For each participant, we computed a variance weighted threshold by multiplying each α-estimate by the inverse of its SE. The mean was computed for each condition, generating subject-level, variance-weighted mean threshold estimates that indicated the proportion of signal in the stimulus required to detect MID at 80% accuracy.

We also calculated an “S-cone performance decrement” for each participant by subtracting the S-cone variance weighted mean threshold from the achromatic variance weighted mean threshold within each cue type (e.g., achromatic CD – S-cone CD). Values at 0 indicate equal performance regardless of chromaticity. Values above 0 indicate improved performance for the S-cone stimulus. Values below 0 indicate a reduction in sensitivity when the input is S-cone.

### MRI Parameters.

High-resolution anatomical T1-weighted scans [repetition time (TR) = 7.8 ms; echo time (TE) = 3.0 ms; inversion time (TI) = 600 ms; flip angle = 20°; field of view (FOV) = 25.6 × 25.6 cm; matrix size = 256 × 256; voxel resolution = 1.0 × 1.0 × 1.0 mm; 176 coronal slices to cover the whole head] were taken in a separate scanning session and were collected on a 3T SIGNA HDx Excite MRI scanner with an eight-channel whole-head phased-array coil (MRI Devices Corp.). Functional data were collected on the same scanner with a 16-channel half-head phased-array coil (Novamed) to improve SNR in the occipital lobe. Standard gradient-echo echo-planar imaging (EPI) scans included two runs with motion localizer stimuli (TR = 3,000 ms; TE = 30 ms; flip angle = 90°; 124 TRs including four dummy volumes; FOV = 19.2 × 19.2 cm; matrix size = 96 × 96; voxel resolution = 2.0 × 2.0 × 2.5 mm) and seven runs with MID stimuli (TR = 3,000 ms; TE = 30 ms; flip angle = 90°; 114 TRs including four dummy volumes; FOV = 19.2 × 19.2 cm; matrix size = 96 × 96; voxel resolution = 2.0 × 2.0 × 2.5 mm). One high-resolution T1 reference scan with the same slice prescription as the functional data were collected for registration of the EPI data to MNI space (TR = 2,100 ms; TE = 8.6 ms; flip angle = 12°; FOV = 19.2 × 19.2 cm; matrix size = 512 × 512; voxel resolution = 0.38 × 0.38 × 2.5 mm; 39 quasi-axial, contiguous slices oriented along the calcarine sulcus and covering the occipital lobe). fMRI data are available for download at https://openneuro.org/datasets/ds001912/ or by request from the authors.

### fMRI Procedure and Task.

Before scanning, participants completed the isoluminance task. The first two functional scans were motion localizer scans designed to tease apart hMT and hMST from within the hMT+ complex (83, 84). Moving and static stimuli were presented in a blocked design, where the four stimulus conditions (full-field coherent radial motion, coherent radial motion restricted to the left or right hemifield, and static dots) were presented for 12 s each, followed by a 12-s blank fixation-only block. Six stimulus cycles were completed in each fMRI scan (6-min run time).

Following the motion localizers, participants completed seven fMRI runs where MID stimuli were presented. The nine stimulus conditions (CD achromatic, CD achromatic control, CD S-cone, CD S-cone control, IOVD achromatic, IOVD achromatic control, IOVD S-cone, IOVD S-cone control, blank fixation-only) were presented in a rapid event-related design, with interstimulus intervals (ISI) determined using Optseq2 (85). Each stimulus was presented for 3 s, with a cosine ramp to avoid signal transients, and the ISI varied between 3 and 12 s. The fixation cross and two fixation rings were presented throughout the whole scan to encourage stable fixation. There were five repeats of each condition in each run, giving a total of 35 repeats of each stimulus condition across all 7 fMRI runs. Each run took 5 min 42 s.

During all fMRI scans, participants completed a challenging task at fixation to control eye position and the allocation of spatial attention. The fixation cross alternated between two different shades of gray, given by the RGB values [0 0 0] and [0.7 0.7 0.7]. These changes occurred at intervals drawn randomly from a uniform distribution ranging between 1,500 and 7,500 ms. Participants were required to track these subtle changes by pressing alternate buttons on a response pad.

### Mapping ROIs.

ROIs (V1, V2, V3, V4, V3A/B, IPS-0, LO-1, LO-2, hMT, and hMST) were mapped on an individual level using a combination of retinotopic mapping (86, 87) and motion localizers (83, 84). Both techniques are illustrated in Fig. 2. V1, V2, V3 (88–90), V4 (91–94), LO-1, LO-2 (95), V3A/B, and IPS-0 (96–98) were determined based on characteristic phase reversals in response to standard retinotopic mapping stimuli (typically, each voxel’s average response across three to five scans consisting of eight cycles of a rotating checkerboard wedge or an expanding ring) collected in a separate scan session (Fig. 2*A*).

To avoid conflating the stimulus-driven response in V1, V2, and V3 with negative BOLD effects in the periphery (99, 100), we restricted these ROIs to the eccentricity that corresponded to the size of the MID stimuli using a contrast map comparing the BOLD response to all stimulus types against fixation. Restricted ROIs were refined using the eccentricity maps from the retinotopic data to ensure correspondence with the known stimulus size.

Motion-sensitive ROIs were identified using a motion localizer designed to identify the hMT+ complex and segregate it into its hMT and hMST subcomponents (Fig. 2*B*). It was modeled on hMT/hMST localizers described previously (83, 84, 101). Briefly, moving black and white dots on a mean gray background (density 9.9 dots per square degree, smoothed Gaussian profile σ = 0.04°, dot speed 5.3°/s) either filled an annulus extending from 0.5°–11.75° eccentricity, or were constrained to the left or right 120° of the display embedded within a static dot pattern updating at 0.33 Hz. Responses to these motion stimuli were contrasted against responses to a static dot stimulus consisting of randomly selected frames from the full-field motion stimulus, updating at 0.33 Hz. Stimuli were shown for 12 s in a blocked design, where each full cycle of stimuli (full-field motion, left hemifield motion, right hemifield motion, static dots) were interleaved with the blank fixation-only block. There were six stimulus cycles per fMRI run, with the same central fixation task used during the MID scans.

The BOLD response across visual areas was modeled using a GLM. Contrasting the response to full-field motion against static conditions resulted in strong activations in V3A/B, IPS0, and hMT+.

As in earlier visual areas, neurons in hMT receive inputs primarily from the contralateral visual hemifield. However, the receptive fields of neurons in hMST extend into the ipsilateral hemifield. Therefore, these two areas can be dissociated based on their differential responses to ipsilateral motion (84). For example, contrasting left hemifield motion against static resulted in strong activations in hMT+ in the right hemisphere but only in a subset of voxels in the hMT+ complex in the left hemisphere. These left hemisphere voxels were assigned to hMST, whereas the remaining voxels where assigned to hMT. After these subdivisions were made, we refined the borders of motion-sensitive ROIs using each subject’s retinotopic data.

We defined the putative CSM area using Talairach coordinates given in the original paper identifying this region as the site of stereo-motion sensitivity (20). Coordinates were [−42.9 –65.9 1.1] in the left hemisphere and [44.4 –61.9 0.1] in the right hemisphere. We grew a 5-mm spherical ROI centered on these coordinates.

### Whole-Brain Analysis.

fMRI data were processed using a standard FEAT pipeline (v6.00, part of the FMRIB’s Software Library, https://fsl.fmrib.ox.ac.uk/fsl/fslwiki). The first four dummy volumes were deleted to account for initial changes in signal intensity before achieving equilibrium. Nonbrain structures were removed from each functional scan using BET (102), and signal intensity was normalized across each 4D dataset by a multiplicative factor of the grand mean. Motion correction was applied using MCFLIRT (103). The time-series of each voxel was temporal high-pass–filtered to remove slow signal drift (Gaussian-weighted least-squares straight line fitting, σ = 50.0 s) and smoothed using a Gaussian kernel at 3-mm FWHM. To register fMRI data to a standard-space image, the T1-weighted reference scan was skull-stripped and FAST-corrected (104) to correct signal drop-off at the front of the head. This image was aligned to the Montreal Neurological Institute-152 2-mm brain using FLIRT (103, 105) and the resulting transformation matrix was applied to the corresponding EPI datasets.

A GLM with nine predictors for each stimulus type was applied to each 4D dataset using FILM (106) with local autocorrelation correction. Events were convolved with a standard γ-function (3-s std, 6-s lag) to model the BOLD response, and the resulting β-weights gave estimates of each voxel’s response to a particular stimulus. A mixed-effects analysis was carried out to combine data across scans and participants using FILM (107–109) and single group averages were generated. The resulting *z*-statistic images for a predetermined set of contrasts were cluster corrected at a significance level of *P* < 0.050.

### ROI Analysis.

For the individual-level ROI analysis, data were processed in mrVista (https://web.stanford.edu/group/vista/cgi-bin/wiki/index.php/Software; Vista Lab, Stanford University) and Matlab 8.5.0 (2015a; The MathWorks). Four dummy volumes were discarded from the fMRI time course, and motion correction was carried out within and between scans. fMRI data were aligned to a high-resolution anatomical scan taken in a separate scan session, using the FAST-corrected and BET-extracted reference anatomical scan as an intermediate step. Alignment between the reference anatomical scan and the high-resolution T1 was achieved using the Nestares algorithm (110). For volume- and surface-based reconstructions, gray and white matter segmentations of the high-resolution T1 scans were carried out using automated algorithms implemented in Freesurfer v5.3.

A GLM analysis was carried out on gray-layer voxels by convolving event sequences for nine different stimulus types with a “difference of Gammas” (from the SPM 8 toolbox, https://www.fil.ion.ucl.ac.uk/spm/) hemodynamic response function (3-s std, 6-s lag) and fitting the modeled time course to the time course of each voxel. This yielded nine β-weights corresponding to nine stimulus types for each voxel. After ROI definition, the β-weights from each voxel were extracted. To generate estimates for the responses to specific stimulus types, responses to control stimuli were subtracted from responses to MID stimuli, yielding estimates for responses to achromatic CD (achromatic CD – achromatic CD control), achromatic IOVD (achromatic IOVD – achromatic IOVD control), S-cone CD (S-cone CD – S-cone CD control), and S-cone IOVD (S-cone IOVD – S-cone IOVD control) in each participant and each ROI. The GLM variance explained for each voxel in each ROI was extracted in a similar way, and data from participants where the mean variance explained across ROIs was less than 5% were discarded (*n* = 6 of a total of 17).

## Supplementary Material

Supplementary File

Supplementary File

Supplementary File

Supplementary File

Supplementary File
