# Supplementary material for: Asymmetries between achromatic and chromatic extraction of 3D motion signals
Source: Proc Natl Acad Sci U S A. 2019 Jun 17;116(27):13631–40. doi: 10.1073/pnas.1817202116 (PMC6612918; doi:10.1073/pnas.1817202116)
Supplement: Supplementary File [file pnas.1817202116.sapp.pdf]

PNAS
www.pnas.org

Supplementary Information for

Asymmetries between achromatic and chromatic extraction of 3D motion signals

Milena Kaestner, Ryan T. Maloney, Kirstie H. Wailes-Newson, Marina Bloj, Julie M. Harris, Antony B. Morland & Alex R. Wade

Milena Kaestner
Email: milenak@stanford.edu

This PDF file includes:

Supplementary text
Figs. S1 to S2
Captions for movies S1 to S4

Other supplementary materials for this manuscript include the following:

Movies S1 to S4

www.pnas.org/cgi/doi/10.1073/pnas.1817202116

Supplementary Information Text

Equating the perceptual salience of achromatic and S-cone stimuli. In a pilot study, participants (N=5) were asked to detect the direction of MID in a set of achromatic and isoluminant S-cone CD and IOVD stimuli similar to those used in the main experiment. The stimuli were oscillating sinusoidally at a rate of 1Hz. Each stimulus was presented for 0.5s, equating to ½ sine wave of MID. Motion could be either a 'bounce towards' or a 'bounce away' from fixation. The stimulus was 100% coherent (all dots contributed to the MID signal) and isolated the CD or the IOVD cue in a manner similar to methods discussed in the main paper. A Bayesian staircasing procedure adjusted the Michelson contrast of the stimulus. Each participant completed 5 runs of each stimulus condition, where each run consisted of 10 practice trials and 90 testing trials with no feedback given. Data from each run were individually fit with a Weibull function. Across-experiment averages were computed using a variance weighted estimate: The threshold estimate at 75% correct was weighted by the standard error of the estimate and averaged across all 5 trials, producing a variance-weighted mean threshold estimate indicating the % contrast required to discriminate the direction of MID for each condition and each participant. The mean of these thresholds was calculated across participants and results are plotted below (Figure S1). In general, similar levels of contrast were required for different MID types (CD or IOVD), but participants required roughly 10x more contrast for S-cone stimuli (up to 20%) than they did for achromatic stimuli (between 1 and 2%). Therefore S-cone MID stimuli should be set a factor of 10 higher in contrast to produce the equivalent MID percept to their achromatic counterparts. This is also similar to the levels required to balance the BOLD response in early visual cortex (see V1 responses shown in Supplementary Figure S2). This result provided part of the rationale for contrast-scaling of the stimuli in the main behavioral and fMRI experiments of this paper.

Raw BOLD amplitude responses across visual areas. The raw BOLD responses to all stimulus conditions in all ROIs are plotted in Figure S2. For each participant, voxel beta weights representing the modelled fMRI signal change during different stimulation conditions, as well as during the baseline 'fixation' condition, were extracted in each ROI. A mean beta weight was calculated across voxels for each of these conditions, within each ROI. The mean beta amplitude during the 'fixation' event was subtracted from the mean beta amplitude to all other stimulus events. These were then averaged at the group level. Note that these responses represent the raw amplitude (adjusted for baseline fixation) of the BOLD signal change to each of the 8 stimulus conditions (achromatic CD, achromatic CD control, achromatic IOVD, achromatic IOVD control, S-cone CD, S-cone CD control, S-cone IOVD, and S-cone IOVD control).

Instructions for free-fusible stimuli. Movies S1-S4 are side-by-side, cross-fusible versions of the stimuli used in the main experiments. These movies work in QuickTime media player. When playing these movies, select 'View → Loop' in your video player to keep them on the screen indefinitely while you fuse. The contrasts of all images have been increased to make them robustly visible on a standard display device.

To free view cross-fusible images, sit at a comfortable distance from the screen and allow your eyes to 'cross' so that you see 'double images' (one from each eye). Fixating on a very distant point may help with this. The central stimulus images from each eye can then be seen on top of each other. This 'fused' image will contain the left most image from the right eye and the right-

most image from the left eye. The easiest one to see is S3 where you should be able to observe a clear percept of motion towards and away from you at a rate of about 1.5Hz.
Stop if you feel uncomfortable (headache, eyestrain, nausea).

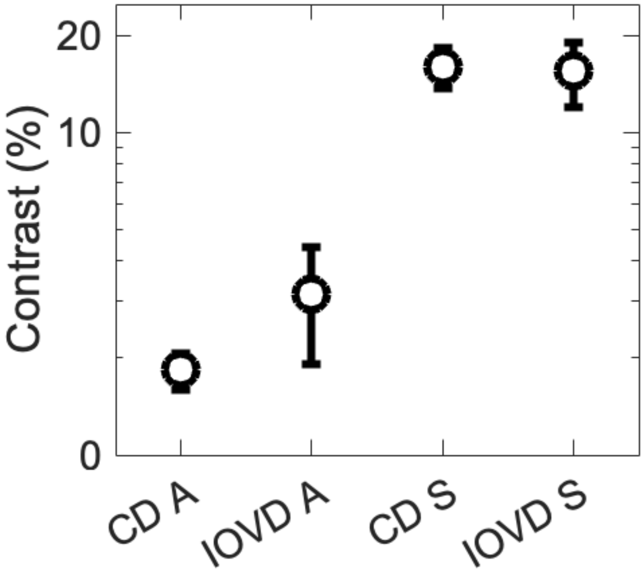

Fig. S1. Contrast (% Michelson) required to discriminate direction in of 3D motion in achromatic and S-cone isolating CD and IOVD stimuli. Error bars indicate ±1 SEM and the Y-axis is log scaled.

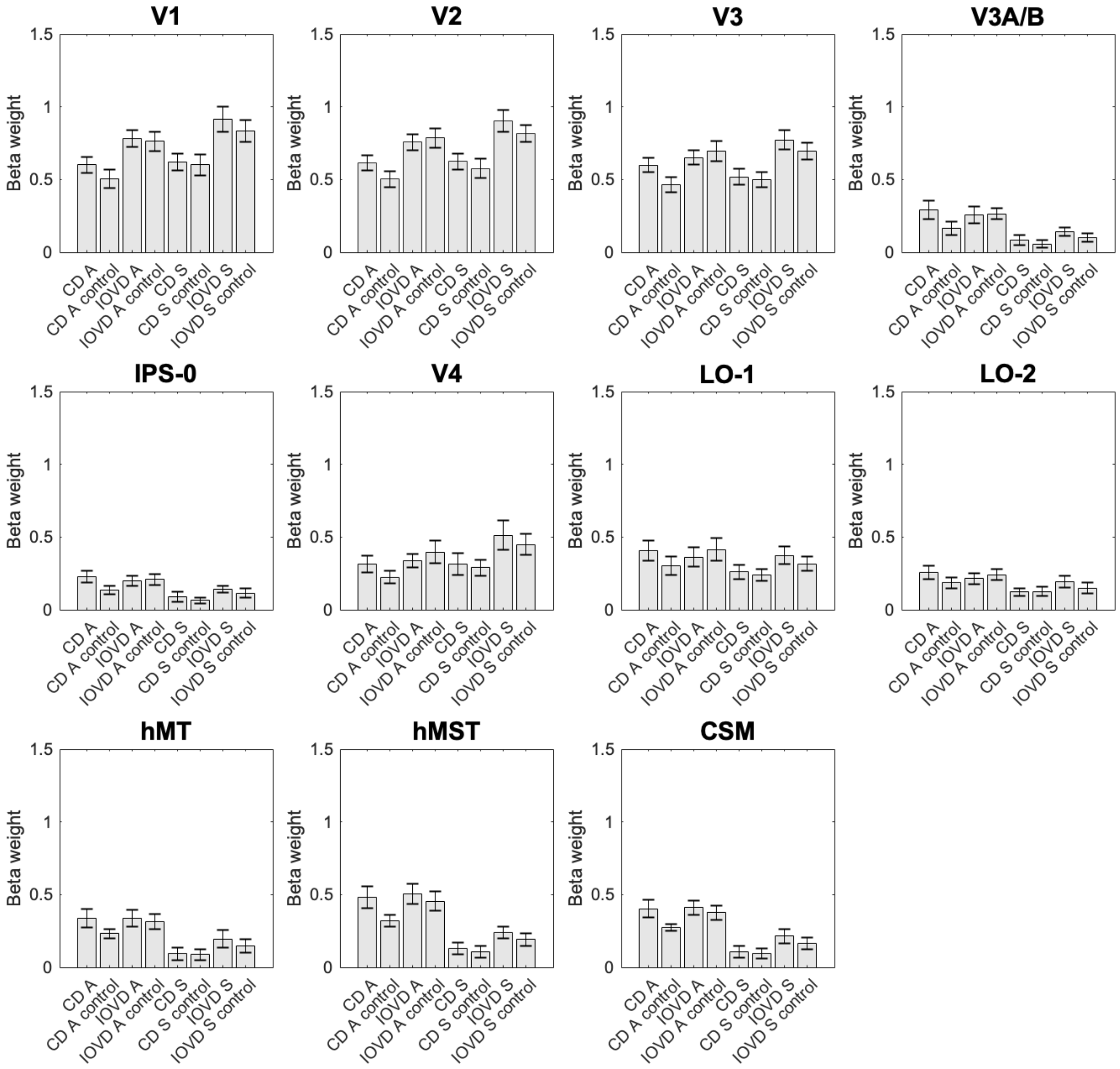

Fig. S2. Raw beta amplitudes, within each ROI and for each stimulus condition. Results were averaged across voxels and across participants. Error bars represent ±1 SEM. The first four bars are responses to achromatic (A) stimuli. The last four bars are responses to S-cone (S) stimuli. Stimulus conditions are shown in this order on the X-axis: CD Achromatic, CD Achromatic control, IOVD Achromatic, IOVD Achromatic control, CD S-cone, CD S-cone control, IOVD S-cone, IOVD S-cone control. All beta amplitudes were adjusted for the response during the baseline fixation condition by subtracting the response during fixation events from the response during stimulation events.

Movie S1. A cross-fusible example of the achromatic CD stimulus. The percept of 3D motion is generated by incrementing the retinal disparity between dot pairs over successive video frames. If you pause the video, you should see the same dot pattern in the left and right half-images. Viewing one half-image gives a percept of flicker. The 3D motion becomes apparent only when both images are fused.

Movie S2. A cross-fusible example of the achromatic IOVD stimulus. The percept of 3D motion is generated by the dot patterns in the left and right images moving in opposite directions. If you pause the video, you should see that the dot patterns in both half-images are different – this is because we eliminated spurious binocular matches to avoid conflating the IOVD cue with CD signals. The 3D motion becomes apparent only when both images are fused. Looking at just one half-images generates a percept of 2D motion.

Movie S3. A cross-fusible example of the S-cone CD stimulus. The percept of 3D motion is generated by incrementing the retinal disparity between dot pairs over successive video frames. If you pause the video, you should see the same dot pattern in the left and right half-images. Viewing one half-image gives a percept of flicker. The 3D motion becomes apparent only when both images are fused.

Movie S4. A cross-fusible example of the S-cone IOVD stimulus. The percept of 3D motion is generated by the dot patterns in the left and right images moving in opposite directions. If you pause the video, you should see that the dot patterns in both half-images are different – this is because we eliminated spurious binocular matches to avoid conflating the IOVD cue with CD signals. The 3D motion becomes apparent only when both images are fused. Looking at just one half-images generates a percept of 2D motion.
